# Association between weight status and migraine in the paediatric population: a systematic review and meta-analysis

**DOI:** 10.3389/fneur.2023.1225935

**Published:** 2023-11-14

**Authors:** Carlos Quispe-Vicuña, David R. Soriano-Moreno, Abraham De-Los-Rios-Pinto, Luz A. Díaz-Ledesma, Daniel Fernandez-Guzman, Kevin Pacheco-Barrios, Carlos Alva-Diaz

**Affiliations:** ^1^Sociedad Científica de San Fernando, Universidad Nacional Mayor de San Marcos, Lima, Peru; ^2^Red de Eficacia Clínica y Sanitaria (REDECS), Lima, Peru; ^3^Unidad de Investigación Clínica y Epidemiológica, Facultad de Medicina, Universidad Peruana Unión, Lima, Peru; ^4^Escuela Profesional de Medicina Humana, Universidad Nacional de San Antonio Abad del Cusco, Cusco, Peru; ^5^Veritas Sociedad Científica de Estudiantes de Medicina, Universidad de San Martín de Porres, Chiclayo, Peru; ^6^Universidad Científica del Sur, Lima, Peru; ^7^Unidad de Investigación para la Generación y Síntesis de Evidencia en Salud, Universidad San Ignacio de Loyola, Lima, Peru; ^8^Neuromodulation Center y Center for Clinical Research Learning, Spaulding Rehabilitation Hospital y Massachusetts General Hospital, Harvard Medical School, Boston, MA, United States; ^9^Departamento de Epidemiología, Escuela de Salud Pública T. H. Chan de Harvard, MA, United States; ^10^Universidad Señor de Sipán, Chiclayo, Peru; ^11^Servicio de Neurología, Departamento de Medicina y Oficina de Apoyo a la Docencia e Investigación (OADI), Hospital Daniel Alcides Carrión, Callao, Peru

**Keywords:** migraine disorders, obesity, overweight, body mass index, thinness

## Abstract

**Introduction:**

An association between weight status and migraine has been previously reported; however, this relationship has only been studied in adults, not in the paediatric population.

**Objective:**

To evaluate the association between weight status and migraine in the paediatric population.

**Methods:**

We searched PubMed/Medline, Scopus, Web of Science, Ovid Medline, and Embase using a cut-off date of May 2023. We included observational studies that evaluated the association between weight status (underweight, overweight, obese, and excess weight) and migraine in the paediatric population (children and adolescents). Normal weight was the comparator. The outcome was migraine (all types, episodic and chronic). We performed meta-analyses using a random-effects model to estimate the pooled effects for each outcome. Sensitivity analysis was performed based on study design and risk of bias (using the Newcastle–Ottawa Scale). Certainty of evidence was assessed using the GRADE approach.

**Results:**

Eight studies (6 cross-sectional, 1 case-control and 1 cohort) covering 16,556 patients were included. The overall certainty of evidence was very low for the association between overweight, obesity, and excess weight with migraine. In the sensitivity analysis, meta-analyses of studies with a low risk of bias found that the overweight population probably had an increased odds of migraine (OR: 1.70; 95% CI: 1.14 to 2.53; *I*^2^ = 32.3%, *p* = 0.224) and that excess weight may increase the odds of migraine (OR: 1.58; 95% CI: 1.06 to 2.35; *I*^2^ = 83.7%, *p* = 0.002). Additionally, cohort and case-control studies found that obesity probably increases the odds of migraine. No studies analysed the association between underweight and migraine.

**Conclusion:**

The associations between overweight, obesity, excess weight and migraine were uncertain, but studies with better methodological quality reported increased odds. Future longitudinal studies with proper confounding control are needed to disentangle their causal relationship.

**Systematic review registration:**

PROSPERO, identifier CRD42021271533.

## 1. Introduction

Migraine is a disabling disorder that affects individuals of all ages (1). Globally, migraine accounts for 5.6% of the burden of disability-adjusted life years and, in general, peaks in the adult population (2). In the last decade, an increase in the prevalence of migraine in childhood and adolescence has been reported, reaching 11% (2, 3). In addition to this, it has been reported that it is in childhood that migraine becomes more frequent and important, which would make childhood a population of special concern (4). The development of headaches in children may be related to many factors, such as genetics, hormones, stress, diet, medication, and dehydration (5).

Factors such as comorbidities, diet, harmful habits, lifestyle, and weight status are associated with the development of migraine in the adult population (6, 7). Additionally, compared with normal weight, obesity, overweight and underweight have been shown to increase the risk of migraine by 27%, 8%, and 13%, respectively (8). Genetic, metabolic-endocrine components such as insulin resistance, and inflammatory components are postulated to be related to the greater presence of tumour necrosis factor (TNF) and interleukin-6 (IL-6), which are increased in individuals with obesity and overweight (9, 10). In addition, changes in body composition—either an increase or decrease—alter the production and secretion of molecules synthesized by adipocytes (e.g., adipocytokines, pro-inflammatory cytokines, and sex hormones) (8, 11), which are related to migraine at the level of the central and peripheral pathways (11).

Hence, the weight status in children could be an important modifiable risk factor for paediatric migraine and the study of this association could serve to understand the complex multifactorial etiology of migraine. However, some studies of children and adolescents report variable results regarding the relationship between weight status and migraine (7, 12–15); some show that being overweight and/or obese is associated with a greater occurrence of migraine (12, 14, 15), and others do not (13). The controversy about this association impedes the implementation of populational strategies that synergies the efforts for preventing both obesity and migraine in children and adolescents. Therefore, herein, we study the association between underweight, overweight and obesity and migraine in the paediatric population.

## 2. Materials and methods

This systematic review and meta-analysis were conducted in accordance with the Preferred Reporting Items for Systematic Reviews and Meta-Analyses (PRISMA) guidelines (16) (Supplementary Table S1). The protocol of this review was registered in PROSPERO (Code: CRD42021271533).

### 2.1. Information sources and search strategy

Structured searches were performed in PubMed, Scopus, Web of Science, Ovid-Medline, and Embase until 10 May 2023. No restrictions on publication date or language were used. The complete search strategy is presented in Supplementary Table S2. In addition, we searched the references of the included studies to identify potentially eligible studies.

### 2.2. Eligibility criteria

We included cross-sectional, case-control and cohort studies that reported relative risk (RR), hazard ratio (HR), odds ratio (OR), or prevalence ratio (PR) and studies that reported data that allowed estimating these measures between weight status (underweight, overweight and obesity) and migraine in paediatric patients (<18 years). Reviews, letters to the editor, meeting or conference abstracts, editorials, comments, case reports and case series, and articles not available in full text were excluded.

### 2.3. Study selection

Duplicate articles were removed automatically with Endnote and then manually with Rayyan. The authors (AD-L-R-P, LD-L, CQ-V, and DF-G) evaluated the inclusion of the studies on the basis of the title and abstract. The full text of potentially eligible studies was reviewed (AD-L-R-P, LD-L, CQ-V, and DF-G), and those that met the inclusion and exclusion criteria were included. This process was performed independently by two pairs of authors (AD-L-R-P, LD-L, CQ-V, and DF-G), and discrepancies were resolved by consensus or by a third author (DS and CA-D).

### 2.4. Exposure and outcome

Weight status in the paediatric population was measured using body mass index (BMI) percentiles: underweight (<5th percentile), normal weight (5–85th percentile), overweight (85–95th percentile), and obesity (>95th percentile) (17). Excess weight was defined as overweight and/or obesity. For migraine, the criteria of the International Classification of Headache Disorders (ICHD) or other sources (e.g., diagnoses in medical records) were considered (Supplementary Table S3). In addition, migraine type (episodic or chronic) was evaluated.

### 2.5. Data extraction and management

The authors (AD-L-R-P, LD-L, CQ-V, and DF-G) extracted the relevant data from each study using an Excel sheet designed for this review. The following information was collected: study design, country, participant characteristics (age, sex, and BMI), sample size, study setting, migraine diagnostic criteria, and migraine type. This process was carried out independently and in pairs, and discrepancies were resolved by consensus or by a third author (DS and CA-D). Numerical variables presented as medians and interquartile ranges were converted to means and standard deviations. We converted relative risk (RR) to odds ratio (OR) as stipulated in the Cochrane manual, considering an assumed comparator risk of 0.09 (4, 18).

### 2.6. Risk of bias

The Newcastle Ottawa Scale (NOS) was used to assess the methodological quality of the studies. This scale measures three sections: study group selection, comparability among groups and association of the exposure with the outcome of interest. The scale is scored with stars; the higher the number of stars, the higher is the methodological quality. For cross-sectional studies, a maximum of 10 stars can be obtained, and for cohort and case-control studies, a maximum of 9 stars (19) can be obtained. Studies with scores ≥6 were considered to have a low risk of bias (high quality), studies with scores of 4–5 were considered to have a moderate risk of bias, and studies with scores <4 were considered to have a high risk of bias (20). The evaluation was carried out in pairs.

### 2.7. Statistical analysis

The association of interest (underweight, overweight, or obesity and migraine) was evaluated, and the variable excess weight, which included overweight and obesity, was created, given the availability of data. The comparator was the normal weight group. We calculated crude odds ratios (ORs) with 95% confidence intervals (95% CIs) and meta-analysed them using DerSimonian and Laird (21) random effects models. Statistical heterogeneity between studies was assessed using the *I*^2^ statistic. Heterogeneity was defined as low if *I*^2^ < 40%, moderate if *I*^2^ = 40%–80%, and high if *I*^2^ > 80%. Additionally, associations were evaluated by type of migraine (episodic or chronic). Subgroup analyses were performed by study design and sensitivity, i.e., risk of bias classification. STATA version 16.0 (StataCorp LP, College Station, Texas, United States) was used.

### 2.8. Evidence certainty assessment

The Grading of Recommendations Assessment, Development and Evaluation (18) was used to report the certainty of evidence through summary of findings (SoF) tables. The GRADE approach was used to appraise the certainty of evidence for all prioritized outcomes (22). This assessment is based on five domains: study limitations (risk of bias of the studies included), imprecision (sample size and CI), indirectness (generalizability), inconsistency (heterogeneity), publication bias, large magnitude of effect, dose-response gradient and effect of plausible residual confounding as stated in the GRADE handbook (23). As the GRADE criteria are mainly used for systematic reviews of interventions or diagnoses, we used the version adapted for prognostic factors (24, 25). The suitability for our study is available in Supplementary Table S4.

### 2.9. Ethical considerations

This systematic review included published and open information, and no human subjects participated in this review. Thus, no ethics committee approval was needed.

## 3. Results

### 3.1. Selected studies

**Figure 1:**
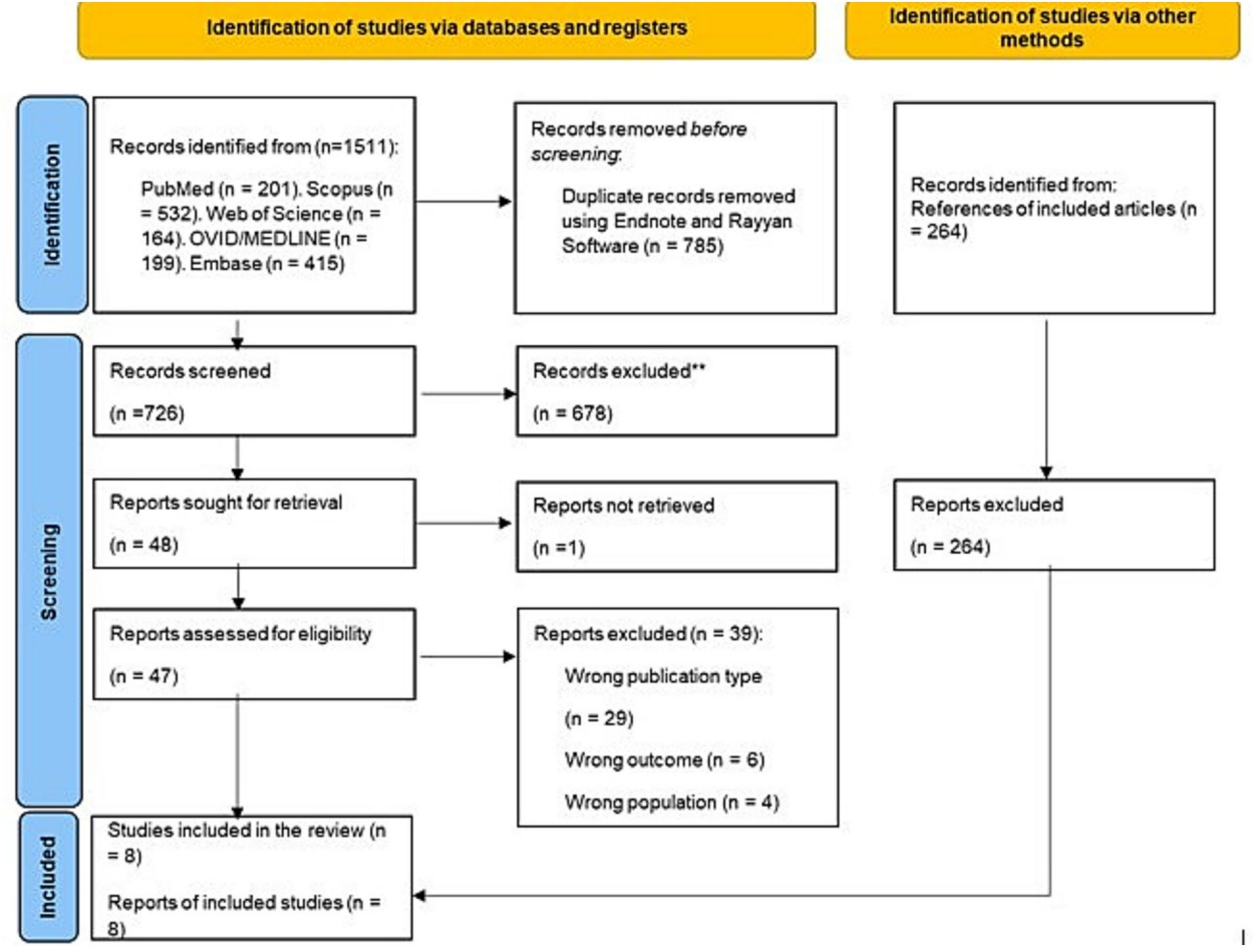
Flow diagram summarizing the literature search and selection process.

A total of 1,511 studies were retrieved, of which 726 remained after removing duplicates. After reading the title and abstract, 678 were eliminated. The full text of the remaining 48 was read. Ultimately, eight studies were included (12–15, 26–29) (Figure 1). The exclusion list and reasons are shown in Supplementary Table S5.

### 3.2. Characteristics of the selected studies

Regarding study design, among the eight studies selected, six were cross-sectional studies (12–15, 26, 29), one was a cohort study (28) and one was a case-control study (27). The studies were conducted in Turkey (12), the United States (26), Israel (13, 14, 29), Taiwan (28), Norway (15) and Italy (27). The total number of participants was 16,556. Five studies were conducted in a hospital setting (13, 14, 26, 27, 29), and three were conducted in a school setting (12, 15, 28) (Table 1).

**Table 1:** Characteristics of the included studies that evaluated the association between weight status and migraine in the paediatric population (*n* = 8).

Author (year)	Country	Study design	Setting, population	Sample size	Population characteristics [age (years) mean ± SD, BMI mean ± SD]	Exposure	Migraine diagnostic criteria and type evaluated in the study
Bektaş et al. (12)-2015	Turkey	Cross-sectional	Community, school population	5,355	Sex, male: 49.4% age: 13.4 ± 2.7 BMI: 19.6 ± 3.1	Overweight and obesity	ICHD II, migraine, probable migraine
Eidlitz-Markus et al. (29)-2015	Israel	Cross-sectional	Hospital, all participants had some type of headache	332	Sex, male: 56.6% age: 11.1 ± 3.7 BMI: 19.52 ± 4.6	Overweight and obesity	ICHD II, migraine, episodic migraine, chronic migraine
Lu et al. (28)-2013	Taiwan	Prospective cohort	Community, school population	3,342	Sex, male: 50.9% age: 13.2 ± 0.5 BMI: NR	Obesity	ICHD II, chronic migraine
Pakalnis and Kring (26)-2012	United States	Cross-sectional	Hospital, all participants had some type of headache	925	Sex, male: 42% age: 12.5 BMI: NR	Overweight and obesity	ICHD II, migraine, probable migraine, episodic or probable episodic migraine, chronic or probable chronic migraine
Pavone et al. (27)-2012	Italy	Case-control	Hospital, all participants had some type of headache	560	Sex, male: 62.5% age: 9.5 ± 3.0 BMI: 16.3 ± 4.6	Obesity	ICHD II, migraine
Pinhas-Hamiel et al. (13)-2008	Israel	Cross-sectional	Hospital, clinic population	273	Sex, male: 39% age: 13.3 ± 2.2 BMI: 1.0 ± 1.3	Overweight and obesity	ICHD II, episodic migraine, chronic migraine
Ravid et al. (14)-2013	Israel	Cross-sectional	Hospital, all participants had some type of headache	181	Sex, male: 44.8% age: 10.1 ± 3.4 BMI: NR	Overweight and obesity	ICHD II, migraine
Robberstad et al. (15)-2010	Norway	Cross-sectional	Community, school population	5,588	Sex, male: 48.0% age: 16.4 ± 0.9 BMI: NR	Overweight/obesity	ICHD II, migraine

SD, standard deviation; BMI, body mass index; NR, not reported; ICHD II, International Classification of Headache Disorders, 2nd edition.

The studies evaluated overweight and obesity; only two evaluated obesity only (27, 28), and one evaluated overweight and obesity as a single exposure (15). No studies evaluated the association between underweight and migraine. All studies used percentiles to classify weight status.

All studies evaluated either episodic or chronic migraine or only migraine in general. To classify migraine type, the studies used the 2nd edition of the International Classification of Headaches (ICHD II) (Table 2). The majority of studies that adjusted for confounding variables found a statistically significant association; the proportion of significant findings was lower in the studies with crude results.

**Table 2:** Association between overweight and obesity and migraine for each included study.

	Exposure^a^	Migraine type	Odds ratio (95% CI)	Confounders
Bektaş et al. (12)	Overweight	All migraine types	1.07 (0.89 to 1.30)	None
			**1.50 (1.11 to 2.01)**	Age, sex
	Obesity		1.29 (0.99 to 1.68)	None
Eidlitz-Markus et al. (29)	Overweight	All migraine types	1.78 (0.85 to 3.72)	None
		Episodic migraine	1.64 (0.75 to 3.57)	
		Chronic migraine	2.05 (0.89 to 4.73)	
	Obesity	All migraine types	0.76 (0.33 to 1.77)	
		Episodic migraine	0.71 (0.28 to 1.81)	
		Chronic migraine	0.84 (0.30 to 2.38)	
Lu et al. (28)	Obesity	Chronic migraine	**2.83 (1.26 to 7.69)**	None
Pakalnis and Kring (26)	Overweight	All migraine types	0.87 (0.64 to 1.18)	None
		Episodic migraine	0.87 (0.63 to 1.19)	
		Chronic migraine	0.87 (0.50 to 1.52)	
	Obesity	All migraine types	0.81 (0.56 to 1.16)	
		Episodic migraine	0.80 (0.55 to 1.16)	
		Chronic migraine	0.86 (0.45 to 1.66)	
Pavone et al. (27)	Obesity	All migraine types	**4.09 (1.36 to 10.98)**	None
Pinhas-Hamiel et al. (13)	Overweight	All migraine types	1.89 (0.58 to 6.12)	None
		Episodic migraine	1.75 (0.28 to 10.85)	
		Chronic migraine	2.00 (0.43 to 9.31)	
	Obesity	All migraine types	1.50 (0.56 to 4.02)	
		Episodic migraine	3.69 (0.99 to 13.79)	
		Chronic migraine	0.11 (0.01 to 2.09)	
Ravid et al. (14)	Overweight	All migraine types	**2.97 (1.47 to 5.99)**	None
			**2.37 (1.21 to 4.67)**	Age, sex
	Obesity		**3.24 (1.30 to 8.11)**	None
			2.29 (0.95 to 5.56)	Age, sex
Robberstad et al. (15)	Overweight and Obesity	All migraine types	**1.60 (1.40 to 2.20)**	Age, sex, smoking, physical activity

OR, odds ratio, RR, risk ratio.

a Compared with normal weight. Values highlighted in bold are statistically significant values.

### 3.3. Risk-of-bias assessment

In the cross-sectional studies, the scores ranged from 4 to 9 out of 10. The domain with the highest score was “outcome,” and the domain with the lowest score was “selection”; the majority of studies (13, 14, 26, 29) were conducted in a hospital setting without community representation of obese and overweight patients. Furthermore, the study by Pavone et al. (27) presented a low response rate for the measurement of the outcome. Additionally, the cohort study by Lu et al. (28) presented little clarity in the measurement of the outcome and did not adjust for confounding variables (Supplementary Table S6).

### 3.4. Overweight and migraine

**Figure 2:**
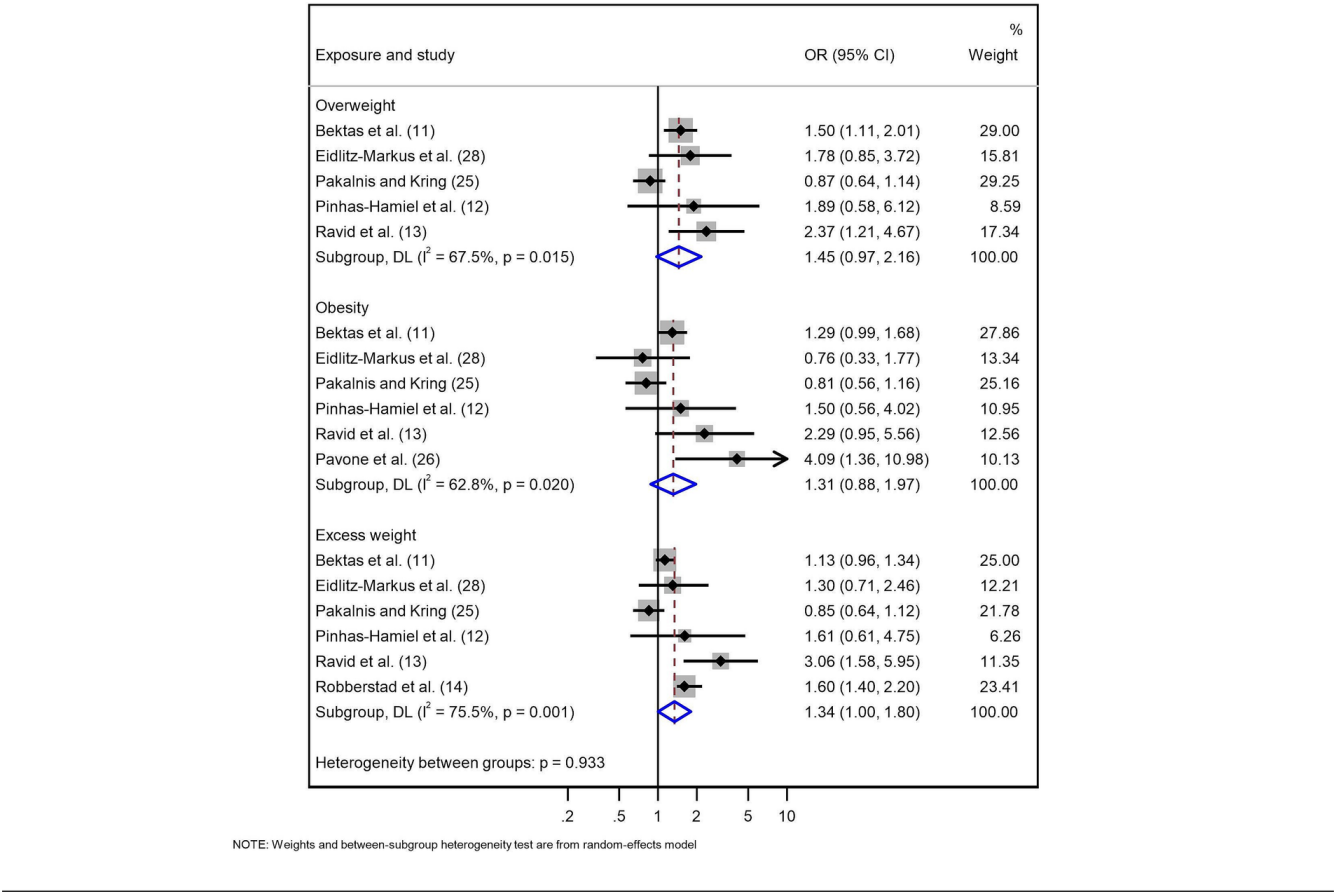
Meta-analysis of the relationship between weight status and migraine in the paediatric population.

Five cross-sectional studies (7,066 patients) (12–14, 26, 29) evaluated the association between overweight and migraine of any type, finding an OR of 1.45 (95% CI: 0.97 to 2.16; *I*^2^ = 67.5%), with very low certainty (⨁◯◯◯). However, by including only the two studies with a low risk of bias (12, 14), there was a greater probability that being overweight is a factor for migraine (OR: 1.70; 95% CI: 1.14 to 2.53; *I*^2^ = 32.3%), with moderate certainty for this estimate (⨁⨁⨁◯) (Table 3; Figure 2; Supplementary Figure S1).

**Table 3:** Summary of findings from Overweight exposure.

Population	Paediatric population				
Exposure	Overweight				
Comparator	Normal weight				
Outcomes	No. of participants (studies)	Relative effect (95% CI)	Absolute effect (95% CI)	Quality of evidence (1)	Comments
All migraine types	7,066 (5 cross-sectional)	OR: 1.45 (0.97 to 2.16)	Not calculated	⨁◯◯◯^a^^,^^b^^,^^c^^,^^d^ very low	In the sensitivity analysis, studies with a low risk of bias were cross-sectional, and the effect increased after adjustment ⨁⨁⨁◯
Episodic migraine	1,063 (3 cross-sectional)	OR: 1.06 (0.68 to 1.64)	13 more per 1,000 (from 80 fewer to 118 more)	⨁◯◯◯^a^^,^^b^^,^^e^ very low	
Chronic migraine	632 (3 cross-sectional)	OR: 1.32 (0.69 to 2.50)	65 more per 1,000 (from 78 fewer to 223 more)	⨁◯◯◯^a^^,^^b^^,^^e^ very low	

a Study design issues: meta-analysis includes cross-sectional studies.

b Very serious risk of bias: more than 50% of the studies present a moderate risk of bias.

c Serious inconsistency: *I*^2^between 40% and 80%.

d Serious imprecision: confidence intervals include the null effect and the 1.25 effect size.

e Very serious imprecision: confidence intervals include the 0.75 and 1.25 effects sizes.

In addition, three cross-sectional studies (13, 26, 29) evaluated the association between overweight and episodic migraine (OR: 1.06; 95% CI: 0.68 to 1.64; *I*^2^ = 22.7%) and chronic migraine, finding a trend towards a greater association with the latter (OR: 1.32; 95% CI: 0.69 to 2.50; *I*^2^ = 38.6%). However, both results had very low certainty (⨁◯◯◯) (Table 3 and Supplementary Figure S2).

### 3.5. Obesity and migraine

Five cross-sectional studies and one case-control study (7,626 patients) (12–14, 26, 27, 29) evaluated the association between obesity and migraine, finding an OR of 1.31 (95% CI: 0.88 to 1.97; *I*^2^ = 62.8%), with very low certainty (⨁◯◯◯). Considering only the case-control study (560 patients) (27), there was an association (OR: 4.09; 95% CI: 1.36 to 10.98), with moderate certainty (⨁⨁⨁◯). For the three studies with a low risk of bias (12, 14, 27), the estimated OR was 1.46 (95% CI: 0.92 to 2.33; *I*^2^ = 32.8%), with low certainty (⨁⨁◯◯) (Table 4; Figure 2; Supplementary Figures S1, S3).

**Table 4:** Summary of findings from Obesity exposure.

Population	Paediatric population				
Exposure	Obesity				
Comparator	Normal weight				
Outcomes	No. of participants (studies)	Relative effect (95% CI)	Absolute effect (95% CI)	Quality of evidence (1)	Comments
All migraine types	7,626 (5 cross-sectional and 1 case-control)	OR: 1.31 (0.88 to 1.97)	Not calculated	⨁◯◯◯^a^^,^^b^^,^^c^^,^^d^ very low	In the subgroup analysis, the case-control study had no concerns and a large effect ⨁⨁⨁◯. In the sensitivity analysis, studies with a low risk of bias were cross-sectional and case-control with no other concerns ⨁⨁◯◯
Episodic migraine	993 (3 cross-sectional)	OR: 1.06 (0.50 to 2.25)	14 more per 1,000 (from 14 fewer to 199 more)	⨁◯◯◯^a^^,^^b^^,^^c^^,^^e^ very low	
Chronic migraine	3,976 (3 cross-sectional and 1 cohort)	OR: 1.02 (0.44 to 2.41)	Not calculated	⨁◯◯◯^a^^,^^b^^,^^c^^,^^e^ very low	In the subgroup analysis, the cohort study had no other concerns ⨁⨁⨁⨁

a Study design issues: meta-analysis includes cross-sectional studies.

b Very serious risk of bias: more than 50% of the studies present a moderate risk of bias.

c Serious inconsistency: *I*^2^between 40% and 80%.

d Serious imprecision: confidence intervals include the null effect and the 1.25 effect size.

e Very serious imprecision: confidence intervals include the 0.75 and 1.25 effects sizes.

For episodic migraine, for three cross-sectional studies (13, 26, 29), the estimated OR was 1.06 (95% CI: 0.50 to 2.25; *I*^2^ = 60.0%), and for chronic migraine, for three cross-sectional studies (13, 26, 29) and one cohort study (28), the estimated OR was 1.02 (95% CI: 0.44 to 2.41; *I*^2^ = 61.8%); both results had a very low level of certainty (⨁◯◯◯). Considering only the cohort study (28), obesity increased the probability of migraine occurrence (OR: 2.83; 95% CI: 1.15 to 6.99), with high certainty (⨁⨁⨁⨁) (Table 4; Supplementary Figures S2, S3).

### 3.6. Excess weight and migraine

Six cross-sectional studies (12,654 patients) (12–15, 26, 29) evaluated the association between excess weight and migraine, finding an OR of 1.34 (95% CI: 1.00 to 1.80; *I*^2^ = 75.5%), with very low certainty (⨁◯◯◯). When only the three studies with a low risk of bias were included (12, 14, 15), the probability was higher (OR: 1.58; 95% CI: 1.06 to 2.35; *I*^2^ = 83.7%), with low certainty (⨁⨁◯◯) (Table 5; Figure 2; Supplementary Figure S1).

**Table 5:** Summary of findings from Excess weight exposure.

Population	Paediatric population				
Exposure	Excess weight (overweight and obesity)				
Comparator	Normal weight				
Outcomes	No. of participants (studies)	Relative effect (95% CI)	Absolute effect (95% CI)	Quality of evidence (1)	Comments
All migraine types	12,654 (6 cross-sectional studies)	OR: 1.34 (1.00 to 1.80)	Not calculated	⨁◯◯◯^a^^,^^b^^,^^c^ very low	In the sensitivity analysis, studies with a low risk of bias were cross-sectional ⨁⨁◯◯
Episodic migraine	1,364 (3 cross-sectional studies)	OR: 1.13 (0.66 to 1.95)	30 more per 1,000 (from 103 fewer to 159 more)	⨁◯◯◯^a^^,^^c^^,^^d^^,^^e^ very low	
Chronic migraine	884 (3 cross-sectional studies)	OR: 1.00 (0.66 to 1.52)	0 more per 1,000 (from 103 fewer to 102 more)	⨁◯◯◯^a^^,^^c^^,^^d^^,^^e^ very low	

a Study design issues: meta-analysis includes cross-sectional studies.

b Serious risk of bias: 50% of the studies present a moderate risk of bias.

c Serious inconsistency: *I*^2^between 40% and 80%.

d Very serious risk of bias: more than 50% of the studies present a moderate risk of bias.

e Very serious imprecision: confidence intervals include the 0.75 and 1.25 effects sizes.

For episodic migraine, for three cross-sectional studies (13, 26, 29), the estimated OR was 1.13 (95% CI: 0.66 to 1.95; *I*^2^ = 55.5%), and for chronic migraine, the estimated OR was 1.00 (95% CI: 0.66 to 1.52; *I*^2^ = 8.2%); both results had very low levels of certainty (⨁◯◯◯) (Table 5 and Supplementary Figure S2).

### 3.7. Certainty of evidence

The body of evidence of the association presented very low certainty (⨁◯◯◯) due to the cross-sectional design of most studies, the moderate risk of bias in more than 50% of the included studies, the high heterogeneity (*I*^2^ > 40) and imprecision in the confidence intervals of the meta-analysed estimates (Tables 3–5). However, some estimates showed moderate certainty for the association between overweight and migraine; in studies with a cross-sectional design, the estimate increased after adjusting for confounding variables. A case-control study showed a large effect measure (OR >2) between obesity and migraine.

## 4. Discussion

### 4.1. Main findings

In this systematic review, we did not find an association between overweight, obesity or excess weight and migraine in the paediatric population. However, studies with a better design and a lower risk of bias reported that these conditions could be associated with presenting with migraine. No studies evaluated the association between underweight and migraine.

### 4.2. Our findings in context

Two systematic reviews (8, 30) showed that in adults, being overweight is not associated with migraine. Those findings are consistent with the results reported herein. However, studies with a low risk of bias reported that being overweight is probably associated with a greater probability of migraine because the paediatric population could be more susceptible than the adult population to changes in weight status and the development of neurological diseases, considering that these individuals are developing and migraine onset usually occurs in the second or third decade of life (31).

Regarding the relationship between obesity and migraine, a previous systematic review found that in adults, obesity was associated with a higher probability of migraine in studies that adjusted for confounding factors (8). These results are consistent with our findings, i.e., studies with a low risk of bias and better design reported that obesity can increase the probability of migraine. One reason for this similarity could be that the fitted models control for potential confounding variables or spurious relationships between the variables. However, there are reports that obesity is associated with chronic migraine (30); this is also consistent with our findings and explained by chronic inflammation and vascular damage, mechanisms that make migraine more chronic (32).

None of the previous systematic reviews evaluated the association between excess weight (overweight and obesity) and migraine. In our analyses, we observed findings similar to those previously reported, i.e., studies with a low risk of bias reported that excess weight could be associated with migraine. These findings could be explained by the increase in pro-inflammatory cytokines in adipose tissue, for example, TNFα, IL-1 and IL-6, which are associated with the development and chronification of migraine (33). However, further mechanistic explorations are needed to test the role of weight status and more severe and chronic migraine in children.

### 4.3. Risk of bias and certainty

In this systematic review, we found a very low certainty of evidence supporting the association between weight status and migraine type; this very low certainty was due to the inconsistency, imprecision and low methodological quality of the set of selected studies. One reason for the inconsistency could be that the size of the sample or participants per study was not assessed *a priori*. In addition, we found that the low risk of bias was mostly due to inadequate methodology in participant selection, especially in sample size, nonresponse rate, and lack of consecutive or randomized sampling. However, when we analysed only the studies with the best design and lowest risk of bias, the certainty improved. All this suggests that future studies should improve the precision of estimates and control for systematic error to confirm the relationship between weight status and migraine. For this purpose, longitudinal studies with a larger sample size and longer follow-up periods are needed to evaluate the association by adjusting for confounding variables such as age, sex, physical activity, and the consumption of prescription or recreational drugs, family history of cardiovascular conditions and migraine, mental health, and other comorbidities which are important factors that may affect this possible association (15, 27, 28, 34, 35).

### 4.4. Clinical and research applicability

Because our findings suggest a tendency for overweight, obesity and excess weight to be related to migraine in children, the care of paediatric patients should include weight status assessments. The aim is to obtain a weight suitable for age, height and population group and to reduce the occurrence or chronification of migraine and other effects observed in adults (36, 37).

Future studies should evaluate the impact of weight status on migraine, migraine types (episodic or chronic), and the frequency and duration of episodes, similar to what has already been done in the adult population (30, 38). For this purpose, it is necessary to have evidence of the effect of malnutrition, a condition that is still prevalent in low-and middle-income countries, on migraine in the paediatric population (39). Similarly, it is necessary to improve the reporting of weight status through a complete nutritional assessment beyond just considering BMI values because BMI does not adequately differentiate between fat mass and lean mass (40). Another interesting research opportunity to explore the causality of this association is to study the changes in migraine prevalence and characteristics in obese migraineurs that underwent bariatric surgery or intensive weight loss programs. In addition, it should also be noted that no studies were found that evaluated the relationship between migraine and underweight in children, which presents a research opportunity for future studies with better methodological quality to evaluate this association.

### 4.5. Limitations and strengths

Our study has some limitations. First, it was not possible to carry out subgroup analyses by age range, degree of obesity, geography, sex, or migraine characteristics (duration, use of medications, refractoriness to treatment, intensity, etc.) because not enough studies were found with such information. Second, the number of studies evaluated was small and most of them cross-sectional; however, we included all the observational studies found and did not limit the sample to cohort studies. Third, because we did not search the grey literature in local nonindexed journals, it is possible that we did not include some relevant studies; however, we searched the main databases and included all available articles without time or language restrictions. In addition, we used the GRADE approach to assess the certainty of the body of evidence and subgroup and sensitivity analyses to assess possible sources of heterogeneity in our findings.

## 5. Conclusions and recommendations

Studies with a low risk of bias report that being overweight, obese or overweight are possibly associated with the higher occurrence of migraine. However, the current evidence is still insufficient. Well-design and well-powered studies should be carried out. We recommend improving the control of overweight, obesity and excess weight in the paediatric population because doing so could prevent the occurrence or chronification of migraine, in addition to the well-known benefits on other health outcomes.

## Data availability statement

The original contributions presented in the study are included in the article/Supplementary material, further inquiries can be directed to the corresponding author.

## Author contributions

CQ-V, DS, KP-B, and CA-D: article evaluation. CQ-V, DS, AD-L-R-P, LD-L, and DF-G: data extraction, results interpretation, and drafting the article. CQ-V, DS, and CA-D: data analysis. CQ-V, DS, AD-L-R-P, LD-L, DF-G, KP-B, and CA-D: critical revision and final approval of the manuscript: All authors contributed to the article and approved the submitted version.

## References

[1] Safiri S Pourfathi H Eagan A Mansournia MA Khodayari MT Sullman MJM . Global, regional, and national burden of migraine in 204 countries and territories, 1990 to 2019. Pain. (2022) 163:e293–309. doi: 10.1097/j.pain.0000000000002275 34001771

[2] GBD 2016 Headache Collaborators. Global, regional, and national burden of migraine and tension-type headache, 1990–2016: a systematic analysis for the global burden of disease study 2016. Lancet Neurol. (2018) 17:954–76. doi: 10.1016/s1474-4422(18)30322-3 30353868 PMC6191530

[3] Onofri A Pensato U Rosignoli C Wells-Gatnik W Stanyer E Ornello R . Primary headache epidemiology in children and adolescents: a systematic review and meta-analysis. J Headache Pain. (2023) 24:8. doi: 10.1186/s10194-023-01541-0, PMID: 36782182 PMC9926688

[4] Waliszewska-Prosół M Straburzyński M Czapińska-Ciepiela EK Nowaczewska M Gryglas-Dworak A Budrewicz S. Migraine symptoms, healthcare resources utilization and disease burden in a large Polish migraine cohort: results from “Migraine in Poland”-a nationwide cross-sectional survey. J Headache Pain. (2023) 24:40. doi: 10.1186/s10194-023-01575-4, PMID: 37041492 PMC10091674

[5] Leonardi M Grazzi L D'amico D Martelletti P Guastafierro E Toppo C . Global burden of headache disorders in children and adolescents 2007–2017. Int J Environ Res Public Health. (2020) 18:250. doi: 10.3390/ijerph18010250, PMID: 33396281 PMC7795582

[6] The Lancet. Join the Lancet 2020 campaign on child and adolescent health. Lancet. (2020) 395:89. doi: 10.1016/s0140-6736(20)30002-7, PMID: 31929015

[7] Farello G Ferrara P Antenucci A Basti C Verrotti A. The link between obesity and migraine in childhood: a systematic review. Ital J Pediatr. (2017) 43:27. doi: 10.1186/s13052-017-0344-1, PMID: 28270183 PMC5341414

[8] Gelaye B Sacco S Brown WJ Nitchie HL Ornello R Peterlin BL. Body composition status and the risk of migraine: a meta-analysis. Neurology. (2017) 88:1795–804. doi: 10.1212/wnl.0000000000003919, PMID: 28404807 PMC5419981

[9] Verrotti A Di Fonzo A Agostinelli S Coppola G Margiotta M Parisi P. Obese children suffer more often from migraine. Acta Paediatr. (2012) 101:e416–21. doi: 10.1111/j.1651-2227.2012.02768.x, PMID: 22823862

[10] Rainero I Govone F Gai A Vacca A Rubino E. Is migraine primarily a metaboloendocrine disorder? Curr Pain Headache Rep. (2018) 22:36. doi: 10.1007/s11916-018-0691-7 29619630

[11] Peterlin BL Rapoport AM Kurth T. Migraine and obesity: epidemiology, mechanisms, and implications. Headache. (2010) 50:631–48. doi: 10.1111/j.1526-4610.2009.01554.x, PMID: 19845784 PMC3969571

[12] Bektaş Ö Uğur C Gençtürk ZB Aysev A Sireli Ö Deda G. Relationship of childhood headaches with preferences in leisure time activities, depression, anxiety and eating habits: a population-based, cross-sectional study. Cephalalgia. (2015) 35:527–37. doi: 10.1177/0333102414547134, PMID: 25149505

[13] Pinhas-Hamiel O Frumin K Gabis L Mazor-Aronovich K Modan-Moses D Reichman B . Headaches in overweight children and adolescents referred to a tertiary-care center in Israel. Obesity. (2008) 16:659–63. doi: 10.1038/oby.2007.88, PMID: 18239560

[14] Ravid S Shahar E Schiff A Gordon S. Obesity in children with headaches: association with headache type, frequency, and disability. Headache. (2013) 53:954–61. doi: 10.1111/head.12088, PMID: 23574609

[15] Robberstad L Dyb G Hagen K Stovner LJ Holmen TL Zwart JA. An unfavorable lifestyle and recurrent headaches among adolescents: the hunt study. Neurology. (2010) 75:712–7. doi: 10.1212/WNL.0b013e3181eee244, PMID: 20720191

[16] Page MJ Mckenzie JE Bossuyt PM Boutron I Hoffmann TC Mulrow CD . The PRISMA 2020 statement: an updated guideline for reporting systematic reviews. BMJ. (2021) 372:n71. doi: 10.1136/bmj.n71, PMID: 33782057 PMC8005924

[17] Centers for Disease Control and Prevention. BMI for children and teens (2021). Available at: https://www.cdc.gov/obesity/basics/childhood-defining.html (Accessed May 5, 2022)

[18] Schünemann H Vist G Higgins J Santesso N Deeks J Glasziou P . Interpreting results and drawing conclusions (2022). Available at: https://training.cochrane.org/handbook/current/chapter-15 (Accessed September 13, 2022)

[19] Wells GA Shea B O’Connell D Peterson J Welch V Losos M . The Newcastle–Ottawa Scale (NOS) for assessing the quality of nonrandomised studies in meta-analyses. Oxford (2000). https://www.ohri.ca/programs/clinical_epidemiology/oxford.asp (Accessed September 13, 2022).

[20] Ulloque-Badaracco JR Ivan STW Al-Kassab-Córdova A Alarcón-Braga EA Benites-Zapata VA Maguiña JL . Prognostic value of neutrophil-to-lymphocyte ratio in COVID-19 patients: a systematic review and meta-analysis. Int J Clin Pract. (2021) 75:e14596. doi: 10.1111/ijcp.14596, PMID: 34228867 PMC9614707

[21] Dersimonian R Laird N. Meta-analysis in clinical trials. Control Clin Trials. (1986) 7:177–88. doi: 10.1016/0197-2456(86)90046-2 3802833

[22] Balshem H Helfand M Schünemann HJ Oxman AD Kunz R Brozek J . Grade guidelines: 3. Rating the quality of evidence. J Clin Epidemiol. (2011) 64:401–6. doi: 10.1016/j.jclinepi.2010.07.015 21208779

[23] Schünemann H Brożek J Guyatt GAO. Grade handbook (2022). Available at: https://gdt.gradepro.org/app/handbook/handbook.html (Accessed September 13, 2022)

[24] Huguet A Hayden JA Stinson J Mcgrath PJ Chambers CT Tougas ME . Judging the quality of evidence in reviews of prognostic factor research: adapting the grade framework. Syst Rev. (2013) 2:71. doi: 10.1186/2046-4053-2-71, PMID: 24007720 PMC3930077

[25] Foroutan F Guyatt G Zuk V Vandvik PO Alba AC Mustafa R . Grade guidelines 28: use of grade for the assessment of evidence about prognostic factors: rating certainty in identification of groups of patients with different absolute risks. J Clin Epidemiol. (2020) 121:62–70. doi: 10.1016/j.jclinepi.2019.12.023, PMID: 31982539

[26] Pakalnis A Kring D. Chronic daily headache, medication overuse, and obesity in children and adolescents. J Child Neurol. (2012) 27:577–80. doi: 10.1177/0883073811420869 21954426 PMC3777610

[27] Pavone P Rizzo R Conti I Verrotti A Mistretta A Falsaperla R . Primary headaches in children: clinical findings on the association with other conditions. Int J Immunopathol Pharmacol. (2012) 25:1083–91. doi: 10.1177/039463201202500425 23298498

[28] Lu SR Fuh JL Wang SJ Juang KD Chen SP Liao YC . Incidence and risk factors of chronic daily headache in young adolescents: a school cohort study. Pediatrics. (2013) 132:e9–e16. doi: 10.1542/peds.2012-1909 23776112

[29] Eidlitz-Markus T Haimi-Cohen Y Zeharia A. Association of pediatric obesity and migraine with comparison to tension headache and samples from other countries. J Child Neurol. (2015) 30:445–50. doi: 10.1177/0883073814553975, PMID: 25428899

[30] Ornello R Ripa P Pistoia F Degan D Tiseo C Carolei A . Migraine and body mass index categories: a systematic review and meta-analysis of observational studies. J Headache Pain. (2015) 16:27. doi: 10.1186/s10194-015-0510-z, PMID: 25903159 PMC4385329

[31] Bigal ME Liberman JN Lipton RB. Age-dependent prevalence and clinical features of migraine. Neurology. (2006) 67:246–51. doi: 10.1212/01.wnl.0000225186.76323.69 16864816

[32] Westgate CSJ Israelsen IME Jensen RH Eftekhari S. Understanding the link between obesity and headache- with focus on migraine and idiopathic intracranial hypertension. J Headache Pain. (2021) 22:123. doi: 10.1186/s10194-021-01337-0, PMID: 34629054 PMC8504002

[33] Tilg H Moschen AR. Adipocytokines: mediators linking adipose tissue, inflammation and immunity. Nat Rev Immunol. (2006) 6:772–83. doi: 10.1038/nri1937 16998510

[34] Kalarchian MA Marcus MD. Psychiatric comorbidity of childhood obesity. Int Rev Psychiatry. (2012) 24:241–6. doi: 10.3109/09540261.2012.678818 22724645

[35] Peterlin BL Rosso AL Williams MA Rosenberg JR Haythornthwaite JA Merikangas KR . Episodic migraine and obesity and the influence of age, race, and sex. Neurology. (2013) 81:1314–21. doi: 10.1212/WNL.0b013e3182a824f7, PMID: 24027060 PMC3806922

[36] Papetti L Moavero R MaN F Sforza G Tarantino S Ursitti F . Truths and myths in pediatric migraine and nutrition. Nutrients. (2021) 13:2714. doi: 10.3390/nu13082714, PMID: 34444875 PMC8399652

[37] Dasari VR Clark AJ Boorigie ME Gerson T Connelly MA Bickel JL. The influence of lifestyle factors on the burden of pediatric migraine. J Pediatr Nurs. (2021) 57:79–83. doi: 10.1016/j.pedn.2020.12.003, PMID: 33353788

[38] Hatami M Soveid N Lesani A Djafarian K Shab-Bidar S. Migraine and obesity: is there a relationship? A systematic review and meta-analysis of observational studies. CNS Neurol Disord Drug Targets. (2021) 20:863–70. doi: 10.2174/1871527320666210713114840 34259152

[39] Victora CG Christian P Vidaletti LP Gatica-Domínguez G Menon P Black RE. Revisiting maternal and child undernutrition in low-income and middle-income countries: variable progress towards an unfinished agenda. Lancet. (2021) 397:1388–99. doi: 10.1016/s0140-6736(21)00394-9, PMID: 33691094 PMC7613170

[40] Nuttall FQ. Body mass index: obesity, BMI, and health: a critical review. Nutr Today. (2015) 50:117–28. doi: 10.1097/nt.0000000000000092, PMID: 27340299 PMC4890841

