# Supplementary material for: Association between weight status and migraine in the paediatric population: a systematic review and meta-analysis
Source: Front Neurol. 2023 Nov 14;14:1225935. doi: 10.3389/fneur.2023.1225935 (PMC10682819; doi:10.3389/fneur.2023.1225935)
Supplement: Supplementary file 1 [file Data_Sheet_1.docx]

Supplementary Materials for

**ASSOCIATION BETWEEN WEIGHT STATUS AND MIGRAINE IN THE PEDIATRIC POPULATION: A SYSTEMATIC REVIEW AND META-ANALYSIS**

Carlos Quispe-Vicuña1,2, David R. Soriano-Moreno3, Abraham De-Los-Rios-Pinto4, Luz A. Díaz-Ledesma5, Daniel Fernandez-Guzman6, Kevin Pacheco-Barrios7,8,9, Carlos Alva-Diaz2,10,11

# Equally contributing authors

correspondence to: [carlos.alexander.alva@gmail.com](mailto:carlos.alexander.alva@gmail.com)

[Table S1: PRISMA Checklist 2](#_Toc49081573)

[Table S2: Search strategy 4](#_Toc49081574)

[Table S3. Operationalization of migraine classification 6](#_Toc49081575)

[Table S4. GRADE criteria for assessing the certainty of the evidence 7](#_Toc49081576)

[Table S5. List of excluded studies after full-text review…………………………………………………………..10](#_Toc49081577)

[Figure S1. Sensitivity analyses by risk of bias of the relationship between overweight, obesity and excess weight and episodic migraine 13](#_Toc49081579)

Figure S2. Meta-analysis of the relationship between overweight, obesity and excess weight and episodic and chronic migraine………………………………………………………………………………………….14

Figure S3. Subgroup analyses by study design of the association between obesity and migraine and chronic migraine………………………………………………………………………………………………………………18

# Table S1: PRISMA Checklist

| **Section/topic** | **#** | **Checklist item** | **Reported on page #** |
| --- | --- | --- | --- |
| **TITLE** | | |  |
| Title | 1 | Identify the report as a literature review. | 01 |
| **ABSTRACT** | | |  |
| Structured summary | 2 | Provide a structured summary including, as applicable: background; objectives; data sources; study eligibility criteria, participants, and interventions; study appraisal and synthesis methods; results; limitations; conclusions and implications of key findings; | 03 |
| **INTRODUCTION** | | |  |
| Rationale | 3 | Describe the rationale for the review in the context of what is already known about your topic. | 04 |
| Objectives | 4 | Provide an explicit statement of questions being addressed with reference to participants, interventions, comparisons, outcomes, and study design (PICOS). | 05 |
| **METHODS** | | |  |
| Eligibility criteria | 5 | Specify study characteristics (e.g., PICOS, length of follow-up) and report characteristics (e.g., years considered, language, publication status) used as criteria for eligibility, giving rationale. | 05 |
| Information sources | 6 | Describe all information sources (e.g., databases with dates of coverage) in the search and date last searched. | 05 |
| Search | 7 | Present full electronic search strategy for at least one database, including any limits used, such that it could be repeated. | 05 |
| Study selection | 8 | State the process for selecting studies (i.e., screening, eligibility). | 06 |
| Risk of bias in individual studies | 9 | Describe methods used for assessing risk of bias of individual studies (including specification of whether this was done at the study or outcome level). | 07 |
| Risk of bias across studies | 10 | Specify any assessment of risk of bias that may affect the cumulative evidence (e.g., publication bias, selective reporting within studies). | 08 |

| **Section/topic** | **#** | **Checklist item** | **Reported on page #** |
| --- | --- | --- | --- |
| **RESULTS** | | |  |
| Study selection | 11 | Give numbers of studies screened, assessed for eligibility, and included in the review, with reasons for exclusions at each stage, ideally with a flow diagram. | 8 |
| Study characteristics | 12 | For each study, present characteristics for which data were extracted (e.g., study size, PICOS, follow-up period) and provide the citations. | 9 |
| Synthesis of results of individual studies | 13 | For all outcomes considered (benefits or harms), present, for each study: (a) summary of results and (b) relationship to other studies under review (e.g. agreements or disagreements in methods, sampling, data collection or findings). | 10-11 |
| **DISCUSSION** | | |  |
| Summary of evidence | 14 | Summarize the main findings including the strength of evidence for each main outcome; consider their relevance to key groups (e.g., healthcare providers, users, and policy makers). | 11 |
| Limitations | 15 | Discuss limitations at study and outcome level (e.g., risk of bias), and at review-level (e.g., incomplete retrieval of identified research, reporting bias). | 14 |
| **CONCLUSION** | | |  |
| Conclusions | 16 | Provide a general interpretation of the results in the context of other evidence, and implications for future research. | 14 |

Free Sample downloaded from: <https://guelphhumber.libguides.com/c.php?g=213266&p=1406923>

*Adapted from:*  Moher D, Liberati A, Tetzlaff J, Altman DG, The PRISMA Group (2009). Preferred Reporting Items for Systematic Reviews and Meta-Analyses: The PRISMA statement*. PLoS Medicine*, 6(6), e1000097. doi:10.1371/journal.pmed1000097

# Table S2: Search strategy

| **PUBMED** | | |
| --- | --- | --- |
| Search terms | | |
| #1 | "Child"[Mesh] OR Child*[TIAB] OR "Adolescent"[Mesh] OR Adolescen*[TIAB] OR Teen*[TIAB] OR Youth*[TIAB] OR juvenil*[TIAB] OR "Minors"[Mesh] OR "Pediatrics"[Mesh] OR "Minor*"[TIAB] OR "Paediatric*"[TIAB] OR "Pediatric*"[TIAB] OR kid*[TIAB] |  |
| #2 | "Migraine Disorders"[Mesh] OR Migrain*[TIAB] OR Hemicrani*[TIAB] OR (Headache*[TIAB] AND Sick[TIAB]) |  |
| #3 | ("Obesity"[Mesh] OR Obesit*[TIAB] OR "Overweight"[Mesh] OR "Overweight"[TIAB] OR corpulency[tiab] OR “fat overload”[tiab] OR “weight insufficiency”[tiab] OR “Thinness"[Mesh] OR “Thinness"[TIAB] OR Leanness[TIAB] OR Underweight[TIAB]) OR (“body weight”[tiab] AND excess[tiab]) |  |
|  | Total Pubmed results on May 10, 2023 | 201 |
| **SCOPUS** | | |
| #1 | (TITLE-ABS-KEY (child* OR adolescen* OR teen* OR youth* OR juvenil* OR minor* OR pediatric* OR paediatric* OR kid*)) AND (TITLE-ABS-KEY( migrain* OR ( headache* AND sick ) OR hemicrania* )) AND (TITLE-ABS-KEY ( obesit* OR overweight OR thinness OR leanness OR underweight OR ( "body weight" AND excess ) OR corpulency OR "fat overload" OR "weight insufficiency" ) ) |  |
|  | Total SCOPUS results on May 12, 2023 | 532 |
| **WEB OF SCIENCE (WOS)** | | |
| #1 | (TS=(Child* OR Adolescen* OR Teen* OR Youth* OR juvenil* OR minor* OR p$ediatric* OR kid*)) AND (TS=(migrain* OR (headache* AND sick) OR hemicrania*)) AND (TS=(obesit* OR overweight OR thinness OR leanness OR underweight OR ("body weight" AND excess) OR corpulency OR "fat overload" OR "weight insufficiency")) |  |
|  | Total WOS results on May 12, 2023 | 164 |
| **OVID MEDLINE** | | |
| #1 | (Child/ OR Adolescent/ OR Minors/ OR Pediatrics/ OR (Child* OR Adolescen* OR Teen* OR Youth* OR juvenil* OR Minor* OR Paediatric* OR Pediatric* OR kid*).ti,ab.) AND (Migraine Disorders/ OR (Migrain* OR Hemicrani*).ti,ab. OR (Headache* AND Sick).ti,ab.) AND (Obesity/ OR Overweight/ OR Thinness/ OR (Obesit* OR Overweight OR corpulency OR fat overload OR weight insufficiency OR thinness OR leanness OR underweight).ti,ab. OR (body weight AND excess).ti,ab.) |  |
|  | Total OVID MEDLINE results on May 12, 2023 | 199 |
| **EMBASE** | | |
| #1 | ('child' OR 'adolescent' OR 'juvenile' OR pediatric OR 'minor (person)')/exp OR (child* OR adolescen* OR teen* OR youth* OR juvenil* OR minor* OR pediatric* OR paediatric* OR kid*):ti,ab,kw) AND ('migraine'/exp OR ( migrain* OR (headache* AND sick) OR hemicrania*):ti,ab,kw) AND (('obesity' OR 'underweight')/exp OR (obesit* OR overweight OR thinness OR leanness OR underweight OR ( 'body weight' AND excess ) OR corpulency OR "fat overload" OR 'weight insufficiency'):ti,ab,kw) |  |
|  | Total EMBASE results on May 12, 2023 | 415 |
| **Search results summary** | | |
| #1 | May 12, 2023 total retrieved results   - Without duplicates | 1511  726 |
| #2 | Additional resources (cites from previous reviews and systematic reviews) | 0 |
| #1+ #2 | TOTAL SR results  TOTAL SR without duplicates | 1511  726 |

# Table S3. Operationalization of migraine classification

| ALL TYPES | EPISODIC | CHRONIC | PROBABLE |
| --- | --- | --- | --- |
| It was considered as the total number of populations with all types and stages of migraine reported by each study. | Migraine that has had a duration of 0 to 14 headache days a month. | **ICHD I 1988***  **ICHD II 2004:** Migraine headache occurring on 15 or more days per month for more than 3 months in the absence of medication overuse  **ICHD III (beta version) 2013:** Headache occurring on 15 or more days per month for more than 3 months, which has the features of migraine headache on at least 8 days per month.  **ICHD III 2018:** Headache occurring on 15 or more days/month for more than three months, which, on at least eight days/month, has the features of migraine headache | **ICHD I 1988:** Headache attacks which are believed to be a form of migraine, but which do not quite meet the operational diagnostic criteria for any of the forms of migraine  **ICHD II 2004:** Attacks and/or headache missing one of the features needed to fulfil all criteria for a disorder coded above  **ICHD III (beta version) 2013:** Migraine-like attacks missing one of the features required to fulfil all criteria for a subtype of migraine coded above, and not fulfilling criteria for another headache disorder.  **ICHD III 2018:** Migraine-like attacks missing one of the features required to fulfil all criteria for a type or subtype of migraine coded above, and not fulfilling criteria for another headache disorder |

*The guide did not provide an established definition

# Table S4. GRADE criteria for assessing the certainty of the evidence

| **Supplementary material 3. GRADE criteria for assessing the certainty of the evidence.** | | | | | | | | |  |
| --- | --- | --- | --- | --- | --- | --- | --- | --- | --- |
| Decision | Study design | Risk of bias | Inconsistency | | Indirectness | Imprecision | Publication bias | | Rating up |
|  |  |  | Statistical heterogeneity | Clinical heterogeneity |  |  | Statistical criteria | Methodological criteria | Applicable if no other concerns excepting study design |
| Not serious | The MA only includes cohort studies | >75% of the studies included in the MA have low risk of bias (NOS >=6). | I2 <40% | < 25% of participants in studies with clinical heterogeneity. | All studies met the inclusion criteria | The confidence interval of the association measure does not pass through either of the imprecision points (0.75 or 1.25). | No graphical, estimation or methodological publication bias | | Rate up 1 level if the effect is between 2-5 or 0.5-0.2, and rate up 2 levels if the effect is >5 or <0.2. Rate up 1 level if the adjusted effect increases the effect of the association. |
| Serious | The MA includes other designs (cross-sectional or case-control) | 75-50% of the studies included in the MA have low risk of bias (NOS >=6) | I2 40-80% with inconsistency of the direction of the association | >25% of participants in studies with clinical heterogeneity. | Outcome other than PICO | The confidence interval of the association measure passes through one of the imprecision points (0.75 or 1.25) and includes the null value | Evaluation of asymmetry in the Funnel Plot. Use of publication bias statistics (Egger). | Analyze whether the search was exhaustive (language, gray publications) |  |
| Very serious |  | <50% of the studies included in the MA have low risk of bias (NOS >=6). | I2 >80% with inconsistency of the direction of the association | >50% of participants in studies with clinical heterogeneity. | Population or exposure other than PICO | The confidence interval of the association measure passes through both imprecision points (0.75 or 1.25). |  |  |  |
| MA: meta-analysis. NOS: New-Castle Ottawa Scale. | | | | | | | | | |

#

# Table S5. List of excluded studies after full-text review

| **Cite** | **Study** | **Title** | **Reason for exclusion** |
| --- | --- | --- | --- |
| (1) | Neuhauser-2011 | The role of overweight on headache, migraine and chronic pain in children and adolescents | Wrong publication type |
| (2) | Farello-2017 | The link between obesity and migraine in childhood: a systematic review | Wrong publication type |
| (3) | Dasari-2021 | The Influence of Lifestyle Factors on the Burden of Pediatric Migraine | Wrong publication type |
| (4) | Evans-2012 | The association of obesity with episodic and chronic migraine | Wrong publication type |
| (5) | Torres-Ferrus-2016 | Teens: Headache and lifestyle in adolescents | Wrong publication type |
| (6) | Le-2012 | Risk factors of migraine - A co-twin control study | Wrong publication type |
| (7) | Ravid-2013 | Obesity in children with chronic daily headaches: Association with headache type and disability | Wrong publication type |
| (8) | Szperka-2019 | Obesity is associated with increased odds of hospitalization following emergent care for pediatric migraine | Wrong publication type |
| (9) | Parisi-2014 | Obesity and Migraine in Children | Wrong publication type |
| (10) | Eidlitz Markus-2018 | Obesity and Migraine in Childhood | Wrong publication type |
| (11) | Bigal-2012 | Obesity and chronic daily headache | Wrong publication type |
| (12) | Verrotti-2012 | Obese children suffer more often from migraine | Wrong publication type |
| (13) | Ravid-2014 | Migraine & paediatric obesity: a plausible link? | Wrong publication type |
| (14) | Annequin-2005 | Migraine in childhood | Wrong publication type |
| (15) | Giraud-2013 | Migraine and obesity, is there a link? | Wrong publication type |
| (16) | Ray-2010 | Migraine and obesity: cause or effect? | Wrong publication type |
| (17) | Casucci-2015 | Migraine and lifestyle in childhood | Wrong publication type |
| (18) | Russo-2016 | Lifestyle Factors and Migraine in Childhood | Wrong publication type |
| (19) | Walter-2014 | Lifestyle Behaviors and Illness-Related Factors as Predictors of Recurrent Headache in US Adolescents | Wrong publication type |
| (20) | Laino-2016 | Headache, migraine and obesity: an overview on plausible links | Wrong publication type |
| (21) | Oakley-2014 | Headache and obesity in the pediatric population | Wrong publication type |
| (22) | Herman-2012 | Frequent migraines and obesity in children and adolescents | Wrong publication type |
| (23) | Ray-2010 | Design and inception of a study to explore putative links between migraine, appetite behaviours and obesity in children | Wrong publication type |
| (24) | Singh-2010 | Current progress of a study to explore putative links between migraine, appetite behaviours and obesity in children | Wrong publication type |
| (25) | Cankay-2020 | Chronic Headache, Comorbidities, Lifestyle and Treatment modalities in an Adolescent Population | Wrong publication type |
| (26) | Pakalnis-2009 | Chronic daily headache, obesity, and medication overuse in a pediatric headache clinic population | Wrong publication type |
| (27) | Eidlitz-Markus-2010 | Association of obesity and migraine in children | Wrong publication type |
| (28) | Torres-Ferrus-2016 | Teens: Headache and lifestyle in adolescents | Wrong outcome |
| (29) | Walter-2021 | Obesity, Migraine, and Overlapping Comorbidities in a Rural Pediatric Population | Wrong outcome |
| (30) | Hershey-2009 | Obesity in the pediatric headache population: A multicenter study | Wrong outcome |
| (31) | Castro-2013 | Lifestyle, quality of life, nutritional status and headache in school-aged children | Wrong outcome |
| (32) | Lateef-2009 | Headache in a national sample of American children: prevalence and comorbidity | Wrong outcome |
| (33) | Ray-2016 | A Pilot Study of Obesogenic Eating Behaviors in Children With Migraine | Wrong outcome |
| (34) | Tarantino-2020 | Anxiety, Depression, and Body Weight in Children and Adolescents With Migraine | Wrong outcome |
| (35) | Bigal-2006 | Obesity is a risk factor for transformed migraine but not chronic tension-type headache | Wrong population |
| (36) | Kinik-2010 | Obesity and paediatric migraine | Wrong population |
| (37) | Peterlin-2013 | Episodic migraine and obesity and the influence of age, race, and sex | Wrong population |
| (38) | Bigal-2007 | Body mass index and episodic headaches: a population-based study | Wrong population |
| (39) | Kinik-2008 | Obesity is a risk factor for the severity of migraine attacks in childhood | No full text |

# Table S6. Risk of bias of included studies

| Risk of bias of cross-sectional studies | | | | | | | | |  | |
| --- | --- | --- | --- | --- | --- | --- | --- | --- | --- | --- |
| Study | Selection (Max. 5*) | | | | Comparability (Max. 2*) | Outcome (Max. 3*) | | Final score (Max. 10) | |  |
|  | Representativeness of the sample | Sample size | Non-respondents | Ascertainment of the exposure (risk factor) | The subjects in different outcome groups are comparable, based on the study design or analysis. Confounding factors are controlled. | Ascertainment of exposure | Statistical test |  |  |  |
| Pinhas-Hamiel – 2008 | - | - | - | ** | - | ** | - | 4 | |  |
| Robberstad - 2010 | * | - | - | ** | ** | ** | * | 8 | |  |
| Pakalnis - 2012 | - | - | * | ** | - | ** | - | 5 | |  |
| Ravid - 2013 | - | - | - | ** | ** | ** | * | 7 | |  |
| Bektaş - 2015 | * | * | - | ** | ** | ** | * | 9 | |  |
| Eidlitz-Markus - 2015 | - | - | - | ** | - | ** | - | 4 | |  |
| The scale is scored with a minimum score of zero and a maximum of ten stars. Each item has a maximum of one star except for the “Ascertainment of the exposure (risk factor)” and "comparability" items which can have two stars. The higher the number of stars, the lower the risk of bias. | | | | | | | | | |  |

| Risk of bias of cohort study | | | | | | | | | | | | | | | | | | | |  |
| --- | --- | --- | --- | --- | --- | --- | --- | --- | --- | --- | --- | --- | --- | --- | --- | --- | --- | --- | --- | --- |
|  | Selection (Max. 4*) | | | | | | | | | Comparability (Max 2*) | | | Exposure (Max. 3*) | | | | |  | |  |
| Study | Representativeness of the exposed cohort | | | Selection of the non exposed cohort | Ascertainment of exposure | | | Demonstration that outcome of interest was not present at start of study | | Comparability of cohorts on the basis of the design or analysis | | | Assessment of outcome | | Was follow-up long enough for outcomes to occur | Adequacy of follow up of cohorts | | Final score (Max. 9) | |  |
| Lu - 2013 | * | | | * | * | | | - | | - | | | * | * | | | * | | 6 | |
| The scale is scored with a minimum score of zero and a maximum of nine stars. Each item has a maximum of one star except for the "comparability" item which can have two stars. The higher the number of stars, the lower the risk of bias. | | | | | | | | | | | | | | | | | | | |  |
| Risk of bias of case-control study | | | | | | | | | | | | | | | | | | | |  |
| Study | | Selection (Max. 4*) | | | | | | | Comparability (Max 2*) | | Exposure (Max. 3*) | | | | | | |  | |  |
|  |  | Is the case definition adequate? | Representativeness of the cases | | | Selection of Controls | Definition of Controls | | Comparability of cases and controls on the basis of the design or analysis | | Ascertainment of exposure | Same method of ascertainment for cases and controls | | | | Non-Response rate | | Final score (Max. 9) | |  |
| Pavone - 2012 | | * | - | | | * | * | | ** | | * | * | | | | - | | 7 | |  |
| The scale is scored with a minimum score of zero and a maximum of nine stars. Each item has a maximum of one star except for the "comparability" item which can have two stars. The higher the number of stars, the lower the risk of bias. | | | | | | | | | | | | | | | | | | | |  |

## Figure S1. Sensitivity analyses by risk of bias of the relationship between overweight, obesity and excess weight and episodic migraine

## Figure S2. Meta-analysis of the relationship between overweight, obesity and excess weight and episodic and chronic migraine.

## Figure S3. Subgroup analyses by study design of the association between obesity and migraine and chronic migraine.

**References**

1. Safiri S, Pourfathi H, Eagan A, Mansournia MA, Khodayari MT, Sullman MJM, et al. Global, regional, and national burden of migraine in 204 countries and territories, 1990 to 2019. Pain. 2022;163(2):e293-e309.

2. Stovner LJ NE, Steiner TJ, Abd-Allah F, Abdelalim A, Al-Raddadi RM, et al. Global, regional, and national burden of migraine and tension-type headache, 1990-2016: a systematic analysis for the Global Burden of Disease Study 2016. The Lancet Neurology. 2018;17(11):954-76.

3. Wöber-Bingöl C. Epidemiology of migraine and headache in children and adolescents. Current pain and headache reports. 2013;17(6):341.

4. Leonardi M, Grazzi L, D'Amico D, Martelletti P, Guastafierro E, Toppo C, et al. Global Burden of Headache Disorders in Children and Adolescents 2007-2017. International journal of environmental research and public health. 2020;18(1).

5. Lancet T. Join the Lancet 2020 Campaign on child and adolescent health. Lancet (London, England). 2020;395(10218):89.

6. Farello G, Ferrara P, Antenucci A, Basti C, Verrotti A. The link between obesity and migraine in childhood: a systematic review. Italian journal of pediatrics. 2017;43(1):27.

7. Gelaye B, Sacco S, Brown WJ, Nitchie HL, Ornello R, Peterlin BL. Body composition status and the risk of migraine: A meta-analysis. Neurology. 2017;88(19):1795-804.

8. Verrotti A, Di Fonzo A, Agostinelli S, Coppola G, Margiotta M, Parisi P. Obese children suffer more often from migraine. Acta paediatrica (Oslo, Norway : 1992). 2012;101(9):e416-21.

9. Rainero I, Govone F, Gai A, Vacca A, Rubino E. Is Migraine Primarily a Metaboloendocrine Disorder? Current pain and headache reports. 2018;22(5):36.

10. Peterlin BL, Rapoport AM, Kurth T. Migraine and obesity: epidemiology, mechanisms, and implications. Headache. 2010;50(4):631-48.

11. Bektaş Ö, Uğur C, Gençtürk ZB, Aysev A, Sireli Ö, Deda G. Relationship of childhood headaches with preferences in leisure time activities, depression, anxiety and eating habits: A population-based, cross-sectional study. Cephalalgia : an international journal of headache. 2015;35(6):527-37.

12. Pinhas-Hamiel O, Frumin K, Gabis L, Mazor-Aronovich K, Modan-Moses D, Reichman B, et al. Headaches in overweight children and adolescents referred to a tertiary-care center in Israel. Obesity (Silver Spring, Md). 2008;16(3):659-63.

13. Ravid S, Shahar E, Schiff A, Gordon S. Obesity in children with headaches: association with headache type, frequency, and disability. Headache. 2013;53(6):954-61.

14. Robberstad L, Dyb G, Hagen K, Stovner LJ, Holmen TL, Zwart JA. An unfavorable lifestyle and recurrent headaches among adolescents: the HUNT study. Neurology. 2010;75(8):712-7.

15. Page MJ, McKenzie JE, Bossuyt PM, Boutron I, Hoffmann TC, Mulrow CD, et al. The PRISMA 2020 statement: an updated guideline for reporting systematic reviews. BMJ (Clinical research ed). 2021;372:n71.

16. (CDC) CfDCaP. Defining Childhood Weight Status BMI for Children and Teens 2021 [updated 2021/12/03/. Available from: <https://www.cdc.gov/obesity/basics/childhood-defining.html>.

17. Schünemann H, Vist G, Higgins J, Santesso N, Deeks J, Glasziou P, et al. Chapter 15: Interpreting results and drawing conclusions. Cochrane Handbook for Systematic Reviews of Interventions Version. 2020;61.

18. Wells G, Shea B, O'Connell D, Peterson j, Welch V, Losos M, et al. The Newcastle–Ottawa Scale (NOS) for Assessing the Quality of Non-Randomized Studies in Meta-Analysis. ᅟ. 2000;ᅟ.

19. Balshem H, Helfand M, Schünemann HJ, Oxman AD, Kunz R, Brozek J, et al. GRADE guidelines: 3. Rating the quality of evidence. Journal of clinical epidemiology. 2011;64(4):401-6.

20. H. S. GRADE handbook for grading quality of evidence and strength of recommendation 2008 [Available from: <https://gdt.gradepro.org/app/handbook/handbook.html>.

21. Huguet A, Hayden JA, Stinson J, McGrath PJ, Chambers CT, Tougas ME, et al. Judging the quality of evidence in reviews of prognostic factor research: adapting the GRADE framework. Systematic reviews. 2013;2:71.

22. Foroutan F, Guyatt G, Zuk V, Vandvik PO, Alba AC, Mustafa R, et al. GRADE Guidelines 28: Use of GRADE for the assessment of evidence about prognostic factors: rating certainty in identification of groups of patients with different absolute risks. Journal of clinical epidemiology. 2020;121:62-70.

23. Pakalnis A, Kring D. Chronic daily headache, medication overuse, and obesity in children and adolescents. Journal of child neurology. 2012;27(5):577-80.

24. Pavone P, Rizzo R, Conti I, Verrotti A, Mistretta A, Falsaperla R, et al. Primary headaches in children: clinical findings on the association with other conditions. International journal of immunopathology and pharmacology. 2012;25(4):1083-91.

25. Lu SR, Fuh JL, Wang SJ, Juang KD, Chen SP, Liao YC, et al. Incidence and risk factors of chronic daily headache in young adolescents: a school cohort study. Pediatrics. 2013;132(1):e9-e16.

26. Eidlitz-Markus T, Haimi-Cohen Y, Zeharia A. Association of pediatric obesity and migraine with comparison to tension headache and samples from other countries. Journal of child neurology. 2015;30(4):445-50.

27. Ornello R, Ripa P, Pistoia F, Degan D, Tiseo C, Carolei A, et al. Migraine and body mass index categories: a systematic review and meta-analysis of observational studies. The journal of headache and pain. 2015;16:27.

28. Tilg H, Moschen AR. Adipocytokines: mediators linking adipose tissue, inflammation and immunity. Nature reviews Immunology. 2006;6(10):772-83.

29. Kalarchian MA, Marcus MD. Psychiatric comorbidity of childhood obesity. International review of psychiatry (Abingdon, England). 2012;24(3):241-6.

30. Peterlin BL, Rosso AL, Williams MA, Rosenberg JR, Haythornthwaite JA, Merikangas KR, et al. Episodic migraine and obesity and the influence of age, race, and sex. Neurology. 2013;81(15):1314-21.

31. Hatami M, Soveid N, Lesani A, Djafarian K, Shab-Bidar S. Migraine and Obesity: Is There a Relationship? A Systematic Review and Meta-Analysis of Observational Studies. CNS & neurological disorders drug targets. 2021;20(9):863-70.

32. Papetti L, Moavero R, Ferilli MAN, Sforza G, Tarantino S, Ursitti F, et al. Truths and Myths in Pediatric Migraine and Nutrition. Nutrients. 2021;13(8).

33. Dasari VR, Clark AJ, Boorigie ME, Gerson T, Connelly MA, Bickel JL. The Influence of Lifestyle Factors on the Burden of Pediatric Migraine. Journal of pediatric nursing. 2021;57:79-83.
